# A white-box model for real-time simulation of acid–base balance in blood plasma

**DOI:** 10.1186/s41077-023-00255-2

**Published:** 2023-06-15

**Authors:** Timothy A. J. Antonius, Willem W. L. van Meurs, Berend E. Westerhof, Willem P. de Boode

**Affiliations:** 1 Department of Neonatology, Radboud University Medical Center, Radboud Institute for Health Sciences, Radboudumc Amalia Children’s Hospital, P.O. Box 9101, 6500 HB Nijmegen, The Netherlands; 2 Department of Pulmonary Medicine, Amsterdam Cardiovascular Sciences, Amsterdam UMC, Vrije Universiteit Amsterdam, Amsterdam, The Netherlands; 3 Consultant in Simulations, 11, Impasse des Balas, 65700 Lahitte-Toupière, France

## Abstract

Maintaining an optimal acid base is important for the patient. The theory underlying acid–base balance can be challenging for clinicians and educators. These considerations justify creating simulations that include realistic changes to the partial pressure of carbon dioxide, pH, and bicarbonate ion concentration in a range of conditions. Our explanatory simulation application requires a model that derives these variables from total carbon dioxide content and runs in real time. The presented model is derived from the Stewart model, which is based on physical and chemical principles, and takes into account the effects of weak acids and strong ions on the acid–base balance. An inventive code procedure allows for efficient computation. The simulation results match target data for a broad range of clinically and educationally relevant disturbances of the acid–base balance. The model code meets the real-time goals of the application and can be applied in other educational simulations. Python model source code is made available.

**Supplementary Information:**

The online version contains supplementary material available at 10.1186/s41077-023-00255-2.

## Introduction

Maintenance of an optimal acid–base balance is important and can be challenging. It depends on the metabolic and ventilatory status of the subject. Quantitative insight into the complex biochemistry of blood acid–base balance has evolved considerably over the last decades [3, 5, 6, 9], but the mathematical formulation of these insights limits their adoption by practicing clinicians and educators. Explanatory models [8] can contribute to passing on such insights to clinical audiences. They are based on interactive visual representations of underlying mathematical models. Dynamically evolving variables in a number of physiologically and clinically relevant compartments are computed and displayed. They respond in real time to several interventions by the user. In this innovation paper, an acid–base balance model underlying an explanatory model is presented. Such a model will also be more generally applicable in educational simulation. To test the accuracy of our model and code, we formulated the following research questions. The initial question concerns experimental validation: “To what extent does the proposed model code precisely capture the pH, pCO_2_, and bicarbonate ion concentration variations in response to changes in total CO_2_ concentration across a range of clinically and educationally significant disruptions of the acid–base balance?” The second question focuses on performance: “Does the implementation of the model code conform to the real-time simulation constraint of the Explain application?”.

### Model requirements

The variables of primary interest in this context are the partial pressure of carbon dioxide pCO_2_(t), pH(t), and bicarbonate ion concentration [HCO_3_^−^](t) in blood plasma, where “(t)” stands for time dependency. Total carbon dioxide concentration [CO_2_]_Σ_(t) is equal to the sum of the concentrations of dissolved carbon dioxide, bicarbonate ions, and carbonate ions. Concentrations of weak (not fully ionized) acids such as albumin and phosphate should be considered. Strong (fully ionized) ions such as sodium, potassium, calcium, magnesium, chloride, and lactase influence the acid–base balance via the balance of charges in plasma.

**Fig. 1:**
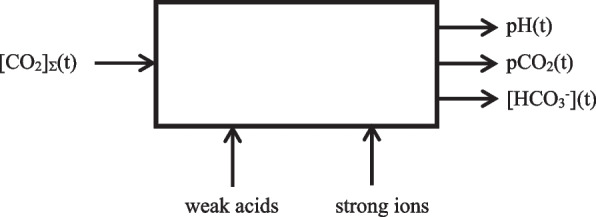
Input–output requirements of the acid–base balance model as integrated in the explanatory model application

The explanatory model uses a carbon dioxide transport model based on [CO_2_]_Σ_(t), rather than on the different ways in which carbon dioxide is stored in blood. Via (total) mass balances, this leads to a simplified model and very efficient code but also introduces the requirement to back calculate the reported blood gases pCO_2_(t), pH(t), and [HCO_3_^−^](t). The first two also play a role in diffusion and autonomic control processes. This leads to the following input–output requirements for the model (Fig. 1).

The explanatory model application calls for an accurate acid–base model linking the above mentioned quantities with a level of model complexity that is adapted to the target audience. Also for educational reasons, and to allow for future expansion, white-box models based on physical principles, such as the ones by Stewart and Reese et al. [3, 4, 6], are preferred over models based on empirical relationships, such as the one by Siggaard-Andersen et al. [5]. Diagnostic and prognostic application of acid–base balance models only requires occasional computation of a single blood gas value. In a real-time explanatory model and other simulation applications, variables are computed frequently and for many compartments. A numerically efficient software implementation of the model is therefore paramount. For both educational and code efficiency reasons, simpler models will be preferred over potentially more accurate but much more complex and computation time-consuming models, such as the ones by Reese et al. and Wolf [3, 4, 9]. To further limit model complexity and optimize real-time performance, the role of erythrocytes and interstitial space in tissues will not be considered for now, but expansion of the model to include such factors should be possible. The original Stewart model [6] fulfills these requirements, albeit with a different input–output configuration (Fig. 1).

In the remainder of this article, the conceptual and mathematical acid–base balance models are described. Specific quantities representing weak acids and strong ions will be introduced. A complete software implementation of the acid–base balance model is made available. Simulation results for a number of respiratory and metabolic disturbances are presented and compared to data from real patients. Run-time performance data are also given. Specific educational applications of this model and code are outlined, but complete development and evaluation of the educational impact of simulators based on the described model are beyond the scope of the present innovation article.

## Methods

### Conceptual model

**Fig. 2:**
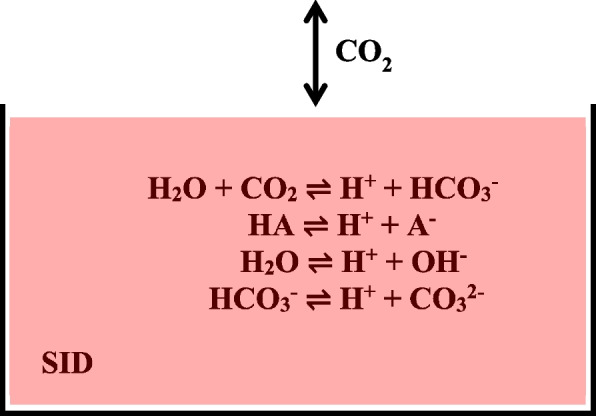
Conceptual representation of the Stewart model [6], adapted from Rees et al. [4]

Figure 2 shows a conceptual model following the Stewart approach [6]. Dissociations of carbonic acid (H_2_CO_3_), nonvolatile weak acids (HA), water (H_2_O), and bicarbonate ions (HCO_3_^−^) all contribute to the concentration of hydrogen ions (H^+^) in a solution. Resulting anions are bicarbonate ions, weak acid anions (A^−^), hydroxide ions (OH^−^), and carbonate ions (CO_3_^2−^), respectively. For the solution to have electrical neutrality, the charges of these ions should balance the charges of strong (completely dissolved) ions, such as sodium (Na^+^), potassium (K^+^), calcium (Ca^2+^), magnesium (Mg^2+^), chloride (Cl^−^), and lactic acid ions (La^−^). The so-called unmeasured anions are indicated by U^−^. The link between electrolyte and acid–base balance is explicitly modeled through the strong ion difference (SID). CO_2_ can be added or removed from the plasma by means of metabolism and minute ventilation. Our model inherits from the Stewart approach that the pH and bicarbonate ion concentrations are dependent on SID, weak acid concentrations, and unmeasured anions.

### Mathematical model

From this section onwards, concentrations will be in mmol/L, charge in mEq/L, and partial pressure in kPa. pH is dimensionless. Constants will be given in units that are consistent with these units. The evolution of total CO_2_ concentration in a compartment, part of the CO_2_ transport model, is based on the mass balance:

1 $$\frac{{d\left[{CO}_{2}\right]}_{\Sigma }\left(t\right)}{dt}=\frac{{f}_{in}\left(t\right)\left\{{\left[{CO}_{2}\right]}_{in}\left(t\right)-{\left[{CO}_{2}\right]}_{\Sigma }\left(t\right)\right\}+{V}_{CO2}(t)}{v(t)}$$

with the total carbon dioxide concentration in the compartment [CO_2_]_Σ_(t), the total concentration of carbon dioxide in the inflow [CO_2_]_in_(t), and the compartment carbon dioxide production V_CO2_(t). The equation also contains the hemodynamic variables compartment volume v(t) and compartment inflow rate f_in_(t). The equation can be expanded to multiple inflow and outflow rates. Total carbon dioxide concentration is the input variable from the CO_2_ transport model to the acid–base model. There is one variable for each site where blood gas values are computed. Total carbon dioxide concentration is equal to the sum of the concentrations of dissolved carbon dioxide, bicarbonate ions, and carbonate:

2 $${\left[{CO}_{2}\right]}_{\Sigma }(t)= {\left[{CO}_{2}\right]}_{di}\left(t\right)+\left[{HCO}_{3}^{-}\right]\left(t\right)+\left[{CO}_{3}^{2-}\right]\left(t\right)$$

The concentration of bicarbonate ions can be obtained from the mass action equation for the dissociation of carbonic acid:

3 $$\left[{HCO}_{3}^{-}\right]\left(t\right)=\frac{{K}_{c}{\left[{CO}_{2}\right]}_{di}(t)}{\left[{H}^{+}\right]\left(t\right)}$$

with the dissociation constant K_c_. For now, the value of the hydrogen ion concentration [H^+^](t) will be considered known. We will come back to this below. Similarly for bicarbonate ions is as follows:

4 $$\left[{CO}_{3}^{2-}\right]\left(t\right)=\frac{{K}_{d}\left[{HCO}_{3}^{-}\right]\left(t\right)}{\left[{H}^{+}\right]\left(t\right)}$$

with the dissociation constant *K*_d_. Substituting Eq. (3) into Eq. (4) and the resulting equation, as well as Eq. (3) into Eq. (2), results in the following:

5 $${\left[{CO}_{2}\right]}_{\Sigma }(t)= {\left[{CO}_{2}\right]}_{di}\left(t\right)+\frac{{K}_{c}{\left[{CO}_{2}\right]}_{di}(t)}{\left[{H}^{+}\right]\left(t\right)}+\frac{{K}_{c}{K}_{d}{\left[{CO}_{2}\right]}_{di}(t)}{{\left[{H}^{+}\right]\left(t\right)}^{2}}$$

From Eq. (5), the concentration of dissolved CO_2_ is computed, based on the given total carbon dioxide concentration and the hydrogen ion concentration:

6 $${\left[{CO}_{2}\right]}_{di}\left(t\right)= \frac{{\left[{CO}_{2}\right]}_{\Sigma }(t)}{1+\frac{{K}_{c}}{\left[{H}^{+}\right]\left(t\right)}+\frac{{K}_{c}{K}_{d}}{{\left[{H}^{+}\right]\left(t\right)}^{2}}}$$

Based on the concentration of dissolved CO_2_ and the hydrogen ion concentration, and using Eq. (3), the bicarbonate ion concentration is computed and based on Eq. (4) the carbonate ion concentration. From the mass equation for dissociation of water, the hydroxide ion concentration is computed as follows:

7 $$\left[{OH}^{-}\right]\left(t\right)=\frac{{K}_{w}\mathrm{^{\prime}}}{\left[{H}^{+}\right]\left(t\right)}$$

with the composite dissociation constant *K*_w_′; the basic water dissociation constant combined with the molar concentration of water. Albumin and phosphate are the main contributors to weak acid anions with an empirically obtained pH dependency given by the following:

8 $$\left[{A}^{-}\right]\left(t\right)=[ALB]\times \left(0.123\times pH\left(t\right)-0.631\right)+[PI]\times \left(0.309\times pH\left(t\right)-0.469\right)$$

with the albumin and phosphate concentrations [ALB] and [PI], respectively, and pH:

9 $$pH\left(t\right)=-log\left\{\left[{H}^{+}\right]\left(t\right)/1000.0\right\}$$

The factor “1000.0” is due to the mmol/L units of [H^+^](t). Charge due to dissociation of acids is as follows:

10 $$\lbrack\mathrm{AC}\rbrack(t)=\left[H^+\right]\left(t\right)-\left[{HCO}_3^-\right]\left(t\right)-\left[A^-\right]\left(t\right)-\left[{OH}^-\right]\left(t\right)-2\left[{CO}_3^{2-}\right]\left(t\right)$$

and the apparent strong ion difference:

11 $${SID}_{app}=\left[{Na}^{+}\right]+\left[{K}^{+}\right]+2\left[{Ca}^{2+}\right]+2\left[{Mg}^{2+}\right]-\left[{Cl}^{-}\right]-\left[{La}^{-}\right]$$

The net charge of the solution results from the sum of the concentrations of all constituent ions:

12 $$\lbrack NC\rbrack\left(t\right)=\left[AC\right]\left(t\right)+{SID}_{app}-\left[U^-\right]$$

with an unmeasured, but presumed constant, anion concentration [U^−^].

Coming back to the hydrogen ion concentration, a root finding procedure assigns successive [H^+^](t) values and goes through the above computations until neutrality is achieved, or NC(t) < *δ*, where *δ* represents the upper limit on the net charge. Within a small physiological interval *pH*_min_ < pH(t) < *pH*_max_, the solution is generally unique. Details of this procedure, as well as the parameters *δ*, *pH*_min_, and *pH*_max_, will be given in the software implementation section. After a pH is found, the partial pressure of carbon dioxide is computed based on the resulting dissolved carbon dioxide:

13 $${pCO}_{2}\left(t\right)=\frac{{\left[{CO}_{2}\right]}_{di}(t)}{\propto }$$

with the solubility coefficient *α*. Table 1 lists the basic, non-patient, and non-condition-specific model parameters in the order in which they appear in the equations.

**Table 1:** Basic parameters of the acid–base model. Dissociation constants at 37 °C

**Name**	**Symbol**	**Value**	**Unit**
Dissociation constant for carbonic acid	*K*_c_	10^−6.10^	mmol/L
Dissociation constant for bicarbonate ions	*K*_d_	10^−10.2^	mmol/L
Composite dissociation constant for water	*K*_w_′	10^−13.6^	mmol/L)^2^
Carbon dioxide solubility coefficient	α	0.23	mmol/(L kPa)

Patient- and condition-specific parameters will be given in the context of the model code verification experiments. The static acid–base model has no state variables.

### Software implementation

The functionality of the Python code listed in the appendix matches (Fig. 1). Arguments of the main function are [CO_2_]_Σ_(t), [ALB], [PI], *SID*_app_, and [U^−^], and it returns pCO_2_(t), pH(t), and [HCO_3_^−^](t). The code is kept as parsimonious as possible to facilitate understanding by readers and use and modification by modelers. It closely matches the model symbols and units. No provisions were made for sophisticated interfacing, handling of errors resulting from unphysiological values, or handling of exceptions raised by the [H +] root-finding routine.

For the real-time Explain application, it is essential that the procedure for finding an [H^+^](t) value is fast and has guaranteed convergence. The Brent root-finding procedure fulfills these requirements [2]. The pH search interval (*pH*_min_, *pH*_max_) was chosen equal to a wide physiological range of 6.5 and 7.8, and the upper limit on the net charge *δ* was set to 10^−8^ mEq/L. A maximum number of iterations of a 100 cycles was specified, but never reached.

Flow of the code as listed in the appendix is as follows: at the code entry point, the value of the input variable and parameters is read in. Then, the model is called, which contains a call to the Brent root-finding routine. This routine calls the [H +] search subroutine, which contains the presented acid–base balance model. After the net charge is minimized, the root finding exits, and output variables are plotted.

### Verification experiment

In a typical blood gas analysis, only pH and pCO_2_ are actually measured, and [HCO_3_^−^] and [CO_2_]_Σ_ are subsequently computed. The main purpose of the presented model is to compute pH, pCO_2_, and [HCO_3_^−^] based on [CO_2_]_Σ_ (Fig. 1). There seems to be one additional unknown quantity and one fewer known quantity. Two factors are critical in the presented solution:

1. The role of the unmeasured anions concentration [U^−^], which is a model parameter, i.e., an additional known quantity. This parameter plays a role in characterizing the specific patient and condition.
2. The inventive procedure of computing the dependent model variables by presuming a known H^+^ concentration and iteratively computing a solution until neutrality is achieved; see the previous section.

Further note that the model is based on the insightful Stewart approach [6], including weak acids and strong ions, and based on explicit physical and chemical principles, as opposed to the more empirical relationships used in blood gas analysis.

To answer the first research question as stated in the introduction, an extensive (*n* = 1864) data set is used. These data consist of anonymized individual blood gas and electrolyte values acquired from all patients in the neonatal intensive care unit of the Radboudumc Amalia Children’s Hospital, Nijmegen, the Netherlands, between Jan. 1, 2020, and Dec. 24, 2021. The model input variable [CO_2_]_Σ_ is part of the data set, as are the patient and condition-specific model parameters [ALB] and [PI]. *SID*_app_ is computed from the reported electrolytes. The model parameter [U^−^] is set using the following equation:

14 $$\left[{U}^{-}\right]= \left[{H}^{+}\right]- \left[{HCO}_{3}^{-}\right]- \left[{A}^{-}\right]- \left[{OH}^{-}\right]-2 \left[{CO}_{3}^{2-}\right]+ {SID}_{app}$$

which follows from the electrical neutrality requirement of the blood gas sample and with [A^−^] computed using Eq. (8). Then model outputs pH, pCO_2_, and [HCO_3_^−^] are computed. The agreement with the target data is calculated using the Bland–Altman test^1^. The computation of [U^−^], and possibly the derivations of [CO_2_]_Σ_ and [HCO_3_^−^], part of the processing of the target data, introduces some circularity in this process. This does not keep us from answering the model code verification question but is the main reason why these experiments are not referred to as a model validation. See the original work by Stewart for model validation [6] and van Meurs [7] for a more detailed discussion of the differences between code verification and model validation. No experiments involving dynamic changes of conditions within a single patient were conducted, and therefore, the time dependency of variable quantities is omitted in this section.

## Results

**Fig. 3:**
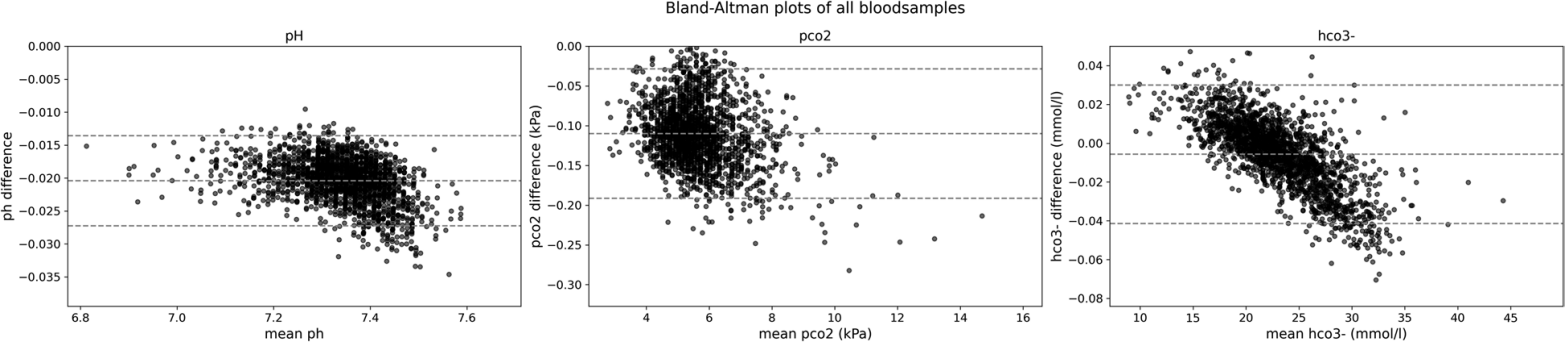
Bland–Altman plots for the agreement between real and predicted values as a function of the average values of pH, pCO_2_, and [HCO_3_.^−^]

Table 2 presents target data and simulation results for single blood gas samples representing typical disturbances. Figure 3 presents a Bland–Altman analysis [1] applied to the full data set. Both Table 2 and Fig. 3 are structured based on the model dependencies.

**Table 2:** Target data and simulation results for a number of disturbances of the acid–base balance, single blood gas sample per disturbance

	**Normal**		**Respiratory acidosis**		**Hyperchloremia**		**Hypoalbuminemia**		**Hyperlactatemia**		**Metabolic acidosis**		
**Input variable**													**Units**
[CO_2_]_Σ_	26.4		22.2		12.9		32.9		16.9		17.6		mmol/L
**Parameters**													**Units**
[ALB]	25		25		20		15		17		16		mmol/L
[PI]	1.64		1.75		1.42		2.4		1.8		2.26		mmol/L
*SID*_app_	35.9		29.7		25.5		42.1		28.1		38.6		mmol/L
[U^−^]	0.0		-0.4		6.0		0.8		4.6		14.1		mmol/L
**Output variables**	Target data	Sim. results	Target data	Sim. results	Target data	Sim. results	Target data	Sim. results	Target data	Sim. results	Target data	Sim. results	
pH	7.40	7.38	7.15	7.14	7.16	7.15	7.49	7.47	7.19	7.18	7.16	7.15	-
pCO_2_	5.60	5.36	8.00	7.67	4.50	4.33	5.70	5.54	5.60	5.36	6.20	5.95	kPa
[HCO_3_^−^]	25.1	25.1	20.4	20.4	11.9	11.9	31.6	31.5	15.6	15.6	16.2	16.2	mmol/L

These results demonstrate that blood gases at baseline and for five clinically relevant acid–base disturbances, or index patients, can be simulated using the model. The data cover a range of values of the independent quantities (input variable and model parameters).

The low mean bias of − 0.0204, − 0.191 kPa, and − 0.005 mmol/l for, respectively, pH, pCO_2_, and HCO_3_^−^ demonstrates that the model code is accurate. The narrow limits of agreement of 0.0068, 0.081 kPa, and 0.035 mmol/l for, respectively, pH, pCO_2_, and HCO_3_^−^ indicate that the model code is precise.

Using the code listed in the Appendix on a Windows personal computer running Windows 10 Professional with an 3.70 GHz Intel core, the average duration of computing the 1864 data points in the above analysis was 0.05 ms per data point. This is sufficient for the Explain real-time application and thereby answering our second research question regarding the real-time constraints of the model. By explicitly programming the Brent root-finding procedure, instead of using the SciPy library, and by using the PyPy implementation of Python 3.7, an even faster implementation can be achieved. With *δ* = 10^−8^ mEq/L, the average number of iterations to get to a solution was 13, with a range of 3 to 18.

## Discussion

Simulated pH and [HCO_3_^−^] closely match the target data. The fact that the model-generated pCO_2_ is systematically lower than measured pCO_2_ could be further explored, but the agreement is considered acceptable for the envisioned educational simulation application. We point again to some circularity in processing of target data and model computations, which limits the use of the experimental data for conclusive model validation, but not for model code verification.

Detailed consideration of the simulated disturbances of Table 2 reinforces model code verification and illustrates potential educational use of the model.

- Conditions leading to an increase in pCO_2_ like respiratory acidosis due to respiratory failure, during mechanical ventilation or other causes of increased CO_2_ production.
- Relative hyperchloremia, which can be caused by excessive administration of chloride containing fluids, such as normal saline, is one of the clinical conditions that is associated with a decreased SID, resulting in metabolic acidosis. It is important to note that the SID only changes when the chloride concentration is high in respect to the sodium concentration.
- Hypoalbuminemia is common in the neonatal intensive care unit, especially in premature neonates. The blood sample demonstrates a mild metabolic alkalosis.
- Hyperlactatemia, caused by anerobic metabolism, is associated with low SID and metabolic acidosis.
- Metabolic acidosis in the listed sample is associated with a high concentration of unmeasured anions, which could be the result of an inborn metabolic defect.

The listed conditions are real, and therefore not pure, disturbances; multiple disturbances may occur simultaneously, and natural compensatory mechanisms play a role. Even in these more challenging conditions, the model behavior is consistent, and simulation results match the target data. The Python code given in the appendix can be used to reproduce the data listed in Table 2. A simulator built around the presented model, such as Explain, would allow for these conditions to be pre-programmed and would add the possibility to make the blood gases evolve in real time via manipulation of therapeutic interventions, such as ventilation or fluid management.

## Conclusion

An original mathematical formulation of the Stewart acid–base balance model [6] was given. An inventive code procedure allows for accurate and efficient computation of the partial pressure of carbon dioxide, pH, and bicarbonate ion concentration, as a function of total carbon dioxide content. The model code implementation was verified by comparing simulation results to clinical target data for a broad range of acid–base disturbances.

## Supplementary Information

**Additional file 1: Appendix.** Model implementation in Python

## Data Availability

All data used for validation of the model is available by sending a request to the corresponding author. The source code of the model is listed in Appendix A.

## Abbreviations

[CO_2_]_Σ_: Total carbon dioxide concentration
A^−^: Weak acid anions
AC: Dissociation of acids
ALB: Albumin
Ca2 +: Calcium
Cl^−^: Chloride
CO_3_^2−^: Carbonate ions
H +: Hydrogen
H_2_CO_3_: Carbonic acid
H2O: Water
HA: Nonvolatile acids
HCO3^−^: Bicarbonate ions
K: Potassium
Kc: Dissociation constant for carbonic acid
Kd: Dissociation constant for bicarbonate ions
K_w′_: Composite dissociation constant for water
La^−^: Lactic acid ions
Mg2 +: Magnesium
Na: Sodium
NC: Net charge
OH^−^: Hydroxide ions
pCO2: Carbon dioxide partial pressure
PI: Phosphates
SID: Strong ion difference
SIDapp: Apparent strong ion
U^−^: Unmeasured anions
*α*: Carbon dioxide solubility coefficient
