# Supplementary material for: A white-box model for real-time simulation of acid–base balance in blood plasma
Source: Adv Simul (Lond). 2023 Jun 15;8:16. doi: 10.1186/s41077-023-00255-2 (PMC10268443; doi:10.1186/s41077-023-00255-2)
Supplement: Supplementary file 1 — Additional file 1: Appendix. Model implementation in Python [file 41077_2023_255_MOESM1_ESM.docx]

**Appendix: Model implementation in Python**

######################################################

# #

# Model code by Timothy Antonius, MD, #

# verification by Willem van Meurs, PhD, Sept. 2022. #

# #

######################################################

# input Python libraries

import math

from scipy import optimize

# set the parameters for the Brent root finding procedure

ph_min = 6.5

ph_max = 7.8

delta = 1e-8

# basic parameters of the acid-base balance model.

Kw = math.pow (10.0, -13.6) * 1000.0 # (mmol/L)^2

Kc = math.pow(10.0, -6.1) * 1000.0 # mmol/L

Kd = math.pow(10.0, -10.22) * 1000.0 # mmol/L

alpha = 0.23 # mmol/L * kPa

# global variables for output (assigned for code simplicity)

ph = 0

pco2 = 0 # kPa

hco3 = 0 # mmol/l

# define H+ search subroutine

def find_hplus(h, tco2, alb, pi, sid_app, u):

global ph, pco2, hco3

cco2 = tco2/(1.0+(Kc/h)+(Kc*Kd)/(math.pow(h,2.0))) # Eq. 6

hco3 = ((Kc * cco2 ) / h) # Eq. 3

cco3 = (Kd * hco3) / h # Eq. 4

oh = Kw / h # Eq. 7

pco2 = cco2 / alpha # Eq. 13

ph = -math.log10(h/ 1000.0) # Eq. 9

a = alb*(0.123*ph-0.631)+pi*(0.309*ph-0.469) # Eq. 8

ac = h - hco3 - a - oh - (2 * cco3) # Eq. 10

nc = ac + sid_app - u # Eq. 12

# return the netcharge to the root finding function

return nc

# define calling routine

def model(tco2,alb,pi,sid_app,u):

# find a [H+] for the given arguments with netcharge = 0

optimize.brentq(f=find_hplus,a=math.pow(10.0,-ph_max)*1000.0,b= math.pow(10.0,-ph_min)*1000.0,xtol=delta,maxiter=100, args=(tco2,alb,pi,sid_app,u))

# entry point and data input

if __name__ == "__main__":

tco2 = float(input("tCO2in mmol/L: "))

sid_app = float(input("SID_app in mEq/L: "))

alb = float(input("Albumin in g/L: "))

pi = float(input("Phosphates in mmol/l: "))

u = float(input("U in mEq/l: "))

# call the model

model(tco2,alb,pi,sid_app,u)

# output

print("pH {}, pCO2 {} kPa, HCO3- {} mmol/L".format(round(ph,2), round(pco2,1),round(hco3,1)))
